# Spin-glass transition in the spin–orbit-entangled *J*_eff_ = 0 Mott insulating double-perovskite ruthenate

**DOI:** 10.1038/s41598-022-06467-2

**Published:** 2022-02-14

**Authors:** Hayato Yatsuzuka, Yuya Haraguchi, Akira Matsuo, Koichi Kindo, Hiroko Aruga Katori

**Affiliations:** 1 Department of Applied Physics, Tokyo University of Agriculture and Technology, Koganei, Tokyo 184-8588 Japan; 2 The Institute for Solid State Physics, The University of Tokyo, Kashiwa, Chiba 277-8581 Japan; 3 Research Center for Thermal and Entropic Science, Graduate School of Science, Osaka University, Toyonaka, Osaka 560-0043 Japan

**Keywords:** Physics, Condensed-matter physics, Magnetic properties and materials

## Abstract

We have successfully synthesized new Ru^4+^ double perovskite oxides SrLaInRuO_6_ and SrLaGaRuO_6_, which are expected to be a spin–orbit coupled *J*_eff_ = 0 Mott insulating ground state. Their magnetic susceptibility is much significant than that expected for a single Ru^4+^ ion for which exchange coupling with other ions is negligible. Their isothermal magnetization process suggests that there are about 20 percent isolated spins. These origins would be the Ru^3+^/Ru^5+^ magnetic defects, while the regular Ru^4+^ sites remain nonmagnetic. Moreover, SrLaGaRuO_6_ shows a spin-glass-like magnetic transition at low temperatures, probably caused by isolated spins. The observed spin-glass can be interpreted by the analogy of a dilute magnetic alloy, which can be seen as a precursor to the mobile *J*_eff_ = 1 exciton as a dispersive mode as predicted.

## Introduction

Recent material investigations have revealed novel phenomena driven by spin–orbit coupling (SOC)^1–20^. The strong SOC splits a *t*_2g_ band into *J* = 3/2 and *J* = 1/2 bands, which results in the realization of the SOC Mott insulating state. For example, the Ir^4+^ ions with the *d*^5^ electron configuration have an effective orbital moment *L* = 1, resulting in a *J*_eff_ = 1/2 pseudospin. Such a spin–orbit-entanglement gives rise to unconventional interaction among pseudospins. In the case of *J*_eff_ = 1/2 pseudospins, the bond-dependent Ising interactions, which have been called the Kitaev interaction in recent years, are realized^1^. Such a realization of the SOC pseudospin state was first observed in layered perovskite Sr_2_IrO_4_^2^. Consequently, it is theoretically predicted that a honeycomb lattice magnet with *J*_eff_ = 1/2 pseudospins is a promising host of a quantum spin liquid (QSL). In the realistic compounds, RuCl_3_ and H_3_LiIr_2_O_6_ exhibit the Kitaev QSL behavior^3–6^.

In contrast, the *d*^4^ electron system (Ru^4+^, Os^4+^, and Ir^5+^) has not been attracted much attention due to an absence of local moments in the ionic ground state. However, *J*_eff_ = 1 excitations become dispersive modes in a crystal due to moderate superexchange (SE) interactions. These mobile spin–orbit excitons may condense in this situation, which results in a magnetically ordered state^7–10^. In order to realize such a state, the exchange interaction must overcome a critical value sufficient to exceed the energy gap *Δ* between the *J*_eff_ = 0 and *J*_eff_ = 1^7,11,12^. Such a condensed state, for which the physicists conceive a terminology—spin–orbit-exciton condensation (SOEC), is analogous to magnon condensation phenomena in a spin dimer system^13^. One factor differentiating the *d*^4^ system from the spin-dimer one is the anisotropy of the strong exchange interaction, which originates from the strong spin–orbit interaction. Therefore, it is expected that a novel condensed phase will be realized.

Although theoretical studies have been enormously advanced to search for anomalous phenomena driven by SOEC, experimental studies have not been carried out due to the lack of model materials. This situation is because the energy scale of *Δ* is too large; the 5*d*^4^ (Ir^5+^ and Os^4+^) compounds should be typically nonmagnetic. Indeed, the weak magnetic anomalies observed in some Ir^5+^ double perovskites are better explained by the Ir^4+^ and Ir^6+^ magnetic defects rather than SOEC^14–21^. Therefore, a SOEC seems to be much less feasible for 5*d* compounds. On the other hand, SOEC is more likely realizable in 4*d*^4^ compounds such as Ru^4+^, where SOC is smaller than Ir^5+^ and is comparable to SE. Moreover, the SOC vs. SE competition can be tuned by a lattice control. Therefore, Ru^4+^ double perovskites would be good model-compounds for a realization of SOEC^22^.

We report the magnetic properties of novel double perovskites SrLaInRuO_6_ and SrLaGaRuO_6_ with Ru^4+^ ion. These deviate significantly from the single-spin susceptibility expected for Ru^4+^ (*J*_eff_ = 0) ions, even though the distance between the magnetic ions is sufficiently large. Furthermore, the magnetization process up to 60 T demonstrates the presence of about 20 percent isolated spins. These behaviors can be explained as originating from the Ru^3+^/Ru^5+^ magnetic defects. Moreover, only SrLaGaRuO_6_ shows a spin-glass transition at *T*_f_ ~ 50 K. We discuss the origin of the observed spin-glass transition from the analogy of the dilute magnetic alloy from the viewpoint of *J*_eff_ = 0 physics in SOC Mott insulators.

## Experimental methods

Polycrystalline samples of SrLa*M*RuO_6_ (*M* = In, Ga) were synthesized by the conventional solid-state reaction from stoichiometric mixtures of SrCO_3_, La_2_O_3_, *M*_2_O_3_ (*M* = In, Ga), and RuO_2_. The obtained samples were characterized by powder X-ray diffraction (XRD) measurements using a diffractometer with Cu *Kα* radiation. The cell and crystal structure parameters were refined using the Rietveld method using rietan-fp version 2.16 software^23^. The temperature dependence of the magnetization was measured using the magnetic property measurement system (MPMS; Quantum Design) equipped in the Institute for Solid State Physics at the University of Tokyo. Magnetization curves up to 60 T were measured using an induction method with a multilayer pulsed magnet at the Institute for Solid State Physics at the University of Tokyo.

## Results

### Crystal structure

**Figure 1:**
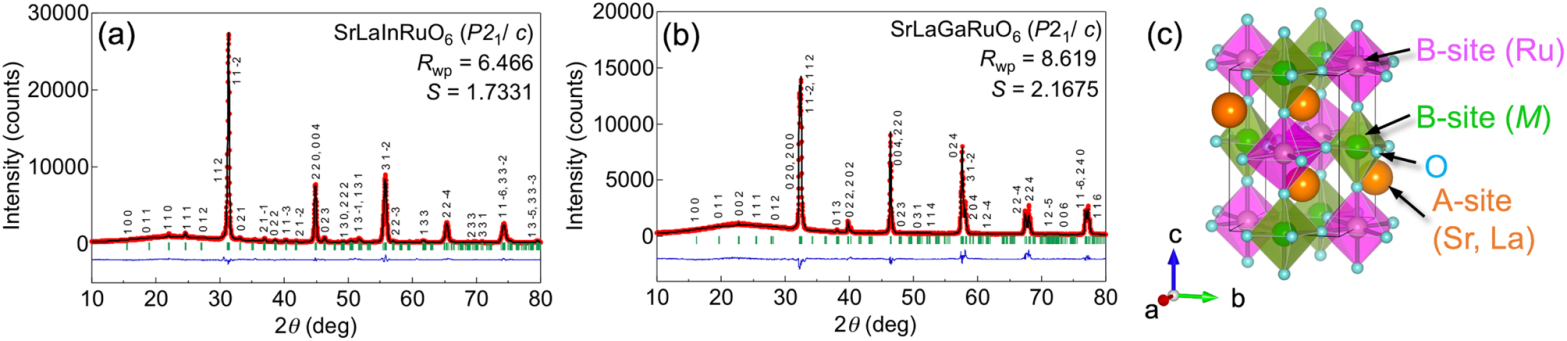
Powder X-ray diffraction patterns of (**a**) SrLaInRuO_6_ and (**b**) SrLaGaRuO_6_. The observed intensities (red), calculated intensities (black), and their differences (blue) are shown. Vertical bars (green) indicate the positions of the Bragg reflections. (**c**) Crystal structure of SrLa*M*RuO_6_ (*M* = In, Ga) obtained from crystal structure parameters refined using the Rietveld method. The vesta program is used for visualization^24^.

Figure 1a and b shows powder XRD patterns from thus-obtained samples. All peaks are indexed to monoclinic unit cells based on the space group of *P*2_1_/c. The Rietveld analysis converged well with the distorted double perovskite structure shown in Fig. 1c and the structural parameters in Table 1. No deviation from the ratio of Sr:La = 1:1 was detected within the experimental error. We estimate the modified tolerance factor *t*_m_ as structural stability in double perovskite using the ionic radii values, yielding *t*_m_ = 0.93834 and 0.98015 in SrLaInRuO_6_ and SrLaGaRuO_6_, respectively. In the condition of *t*_m_ < 1, the double perovskite-type compounds should crystallize a monoclinic structure^25,26^. Our samples certainly satisfy the criterion.

**Table 1:** Crystallographic parameters for SrLaInRuO_6_ and SrLaGaRuO_6_ (both space group: *P*2_1_/*c*) determined from powder X-ray diffraction experiments.

atom	site	*x*	*y*	*z*	*B* (Å)
**SrLaInRuO**_**6**_					
Sr/La	4e	0.5060 (7)	0.5346 (3)	0.252 (1)	0.23 (4)
In	2c	0	1/2	0	1.3 (3)
Ru	2b	1/2	0	0	1.3 (3)
O1	4e	0.229 (7)	0.222 (7)	0.992 (6)	2.7 (2)
O2	4e	0.324 (7)	0.704 (6)	0.963 (4)	2.7 (2)
O3	4e	0.405 (5)	0.988 (2)	0.222 (5)	2.7 (2)
**SrLaGaRuO**_**6**_					
Sr/La	4e	0.4999 (3)	0.498 (3)	0.2492 (9)	0.10 (4)
Ga	2c	0	1/2	0	0.97 (7)
Ru	2b	1/2	0	0	0.97 (7)
O1	4e	0.24 (1)	0.241 (7)	1.007 (6)	2.2 (2)
O2	4e	0.328 (8)	0.676 (5)	1.028 (3)	2.2 (2)
O3	4e	0.50 (2)	0.99 (2)	0.250 (4)	2.2 (2)

The obtained lattice parameters are *a* = 5.6976 (4), *b* = 5.7427 (4), *c* = 8.0669 (5) Å, and *β* = 90.05 (1)º for SrLaInRuO_6_, and *a* = 5.5077 (4), *b* = 5.5538(3), *c* = 7.8210 (3) Å, and *β* = 90.586 (3)º for SrLaGaRuO_6_. *B* is the atomic displacement parameter.

In estimating the valence of the B-site cations at the center of the octahedral *M*O_6_ (*M* = In, Ga) and RuO_6_ in the two double perovskite oxides, we used the bond valence sum *B*_V_ expressed by the following formula^27^,

1 $${B}_{V}={\sum }_{i}^{N}\mathrm{exp}\left(\frac{{R}_{0}-{R}_{i}}{0.37}\right)$$

where *R*_0_ is the empirical bonding parameter, *R*_i_ is the inter-bond cation–anion distance, and *N* is the coordination number. The estimated *B*_V_ values of In/Ga and Ru ions in SrLaInRuO_6_ and SrLaGaRuO_6_ are listed in Table 2, which aligns with the expected values.

**Table 2:** Calculation of the bond valence sum (*B*_V_) for the octahedron in SrLaInRuO_6_ and SrLaGaRuO_6_.

Formula	cation	Bond valence sum
SrLaInRuO_6_	In^3+^	+ 2.838
	Ru^4+^	+ 4.352
SrLaGaRuO_6_	Ga^3+^	+ 2.944
	Ru^4+^	+ 4.056

### Magnetism

**Figure 2:**
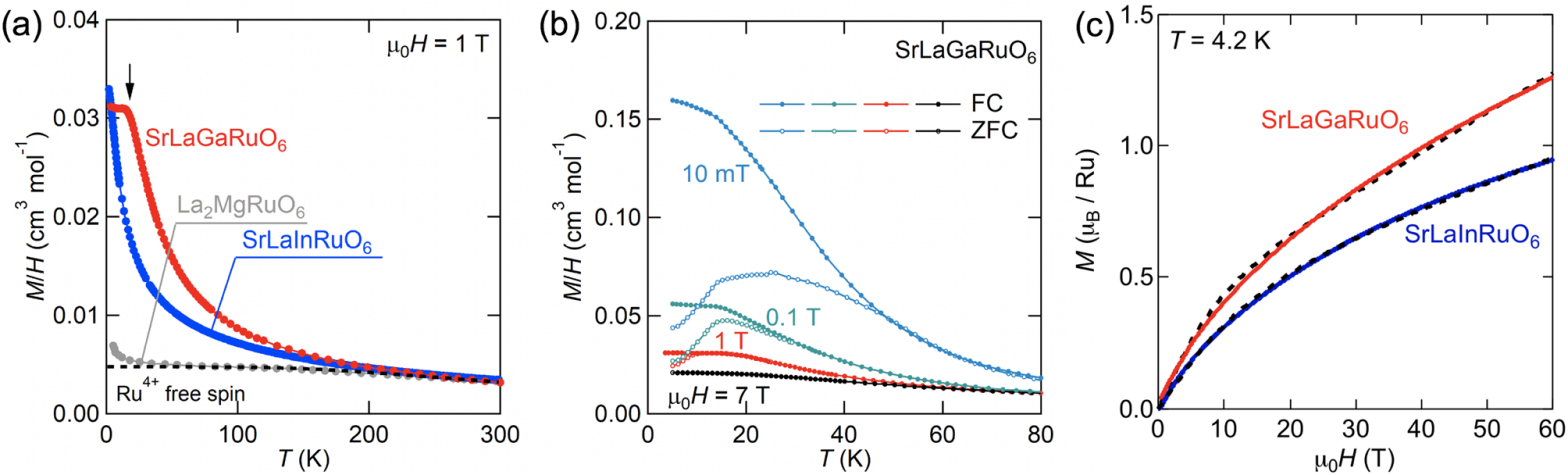
(**a**) Temperature dependence of magnetic susceptibility *M/H* of SrLa*M*RuO_6_ (*M* = In, Ga) and La_2_MgRuO_6_^28,29^ under a magnetic field of 1 T. In this figure, only the results of field cooling (FC) data are shown. The black dotted curve shows the free-spin magnetization of Ru^4+^ ions calculated as described in the text. (**b**) The *M*/*H* curves of SrLaGaRuO_6_ under several magnetic fields. In each field, measurements were conducted upon heating after zero-field cooling (ZFC, open circles) and then upon cooling (FC, closed circles). (**c**) The isothermal magnetization of SrLaInRuO_6_ and SrLaGaRuO_6_ under a magnetic field up to 60 T at 4.2 K. The black dashed lines represent the best fit by Eq. (4) described in the text.

Figure 2a shows the temperature dependence of magnetization *M/H* of SrLa*M*RuO_6_ (*M* = In, Ga) under an applied field of 1 T. For comparison, the *M/H* data of La_2_MgRuO_6_^28,29^ with a similar *d*^4^ electron configuration is displayed. The magnetic response of SrLa*M*RuO_6_ and La_2_MgRuO_6_ is quite different despite the similar electronic state of the Ru^4+^ single ion. When considered from a crystallographic point of view, these compounds are not expected to have strong magnetic interactions because of the significant separation of Ru^4+^ ions. Therefore, it seems strange that the magnetic responses of SrLa*M*RuO_6_ and La_2_MgRuO_6_ are so different. Kotani theoretically predicted the effective magnetic moment of *d*^*n*^ ions (*n* = 1 ~ 5) as a function of electron filling *n*, spin–orbit coupling, temperature, and ligand environment^30^. The effective magnetic moment μ_eff_ (*T*) of low-spin *d*^4^ in an octahedral environment can be expressed as follows,

2 $$\begin{array}{c}{{\mu }_{\mathrm{eff}}}^{2}=\frac{3\left[24+\left(x/2-9\right){e}^{-x/2}+\left(5x/2-15\right){e}^{-3x/2}\right]}{x\left[1+3{e}^{-x/2 }+5{e}^{-3x/2}\right]}\end{array}$$

where *x* = λ/*k*_B_*T*, λ is the spin–orbit coupling interaction^30^, and *k*_B_ is the Boltzmann constant. Thus, the magnetic susceptibility of isolated Ru^4+^ ions χ_calc_ can be expressed as follows,

3 $$\begin{array}{c}{\chi }_{\mathrm{calc}}=\frac{N{{\mu }_{\mathrm{eff}}}^{2}}{3{k}_{B}T}\end{array}$$

The dotted black curve in Fig. 2a represents the χ_calc_ curve calculated using a value of λ = 980 cm^−1^ for Ru^4+^ ions. The λ-value is expected to be smaller than the completely free-ion value of λ = 1400 cm^−1^ determined in the study used Ru^4+^ complexes^31^, which origin of λ-shrinking would be covalency. Note that it is necessary to incorporate the effect of the low symmetry field in order to reproduce the susceptibility in distorted Ru^4+^ double perovskites since Eq. (2) is calculated in the cubic symmetry field. The *M/H* data of La_2_MgRuO_6_ seemingly follows χ_calc_, while those of SrLa*M*RuO_6_ significantly deviate from χ_calc_. This fact indicates that the Ru^4+^ ions in SrLa*M*RuO_6_ are not simply in the *J*_eff_ = 0 ground state.

Both *M/H* data for SrLa*M*RuO_6_ are in the rough agreement above 250 K, but below 250 K, are greatly enhanced compared to the χ_calc_ curve. In Ir^5+^ double perovskites, in which a similar *J*_eff_ = 0 ground state is expected, the *M/H* data of Sr_2_YIrO_6_ shows almost temperature-independent behavior^21^. On the other hand, a similar enhancement in low-temperature *M/H* data is observed in the solid solution system Sr_2-*x*_Ca_*x*_YIrO_6_^21^. Therefore, this increase in magnetization may affect randomness, of which a mechanism will be discussed later.

Moreover, SrLaGaRuO_6_ shows a magnetic anomaly at low temperatures (displayed by an arrow in Fig. 2a), contrasting with no anomaly in SrLaInRuO_6_. Figure 2b expands the low-temperature region under magnetic fields from 0.01 to 1 T. At the lowest field of 0.01 T, the *M/H* data exhibit an apparent thermal hysteresis between the zero-field-cooled (ZFC) and field-cooled (FC) data below *T*_f_ ~ 50 K. This hysteresis is suppressed by increasing the magnetic field and is eventually merged at 7 T. This behavior is a typical feature of spin-glass transition^32^.

### High-field magnetization

Figure 2c shows the isothermal magnetization *M* up to 60 T. The *M-H* curves show convex behavior upward, implying an isolated spin different from the van Vleck magnetism of Ru^4+^ pseudospin *J*_eff_ = 0 state. The origin of the isolated spin will be discussed later. The increase in magnetization at high-field regions is due to the van Vleck paramagnetism.

## Discussion

As described above, the two novel double perovskite ruthenates SrLaInRuO_6_ and SrLaGaRuO_6_ are expected to show a van Vleck magnetism of Ru^4+^ pseudospin *J*_eff_ = 0 state. However, the observed *M/H* is considerably larger than a single Ru^4+^ spin, indicating the deviation from the *J*_eff_ = 0 state. In addition, the isothermal magnetization demonstrates the existence of an isolated spin.

A similar enhancement of magnetization has been reported in highly solid-solution double perovskite iridates Sr_2-*x*_Ca_*x*_YIrO_6_^21^. An X-ray magnetic circular dichroism (XMCD) measurement demonstrates an emergent partial charge disproportionation (PCD) of Ir^5+^ → 0.5Ir^4+^ + 0.5Ir^6+^ due to a site-randomness^21^. In light of this result, similar Ru^3+^/Ru^5+^ magnetic defects possibly occurs in SrLaInRuO_6_ and SrLaGaRuO_6_ due to a similar intrinsic A-site randomness.

The magnetization of isolated spin *M*_iso_ follows a Brillouin function, while the van Vleck term *M*_VV_ should be proportional to *H*. Both terms would contribute to the observed nonlinear behaviors of the isothermal *M*. Here, in order to separate the contributions of the isolated spins and the van Vleck term, we analyze the *M* data with a modified Brillouin function,

4 $$M={M}_{\mathrm{iso}}+{M}_{\mathrm{vv}}={\sum }_{J}{N}_{J}{g}_{J}{\mu }_{B}J\left\{\frac{2J+1}{2J}\mathrm{coth}\left(\frac{2J+1}{2J}\frac{{g}_{J}{\mu }_{B}JH}{{k}_{B}T}\right)-\frac{1}{2J}\mathrm{coth}\left(\frac{1}{2J}\frac{{g}_{J}{\mu }_{B}JH}{{k}_{B}T}\right)\right\}+{\chi }_{\mathrm{vv}}H$$

where *N*_*J*_ represents a scaling factor to account for a finite number of paramagnetic free spins, g_J_ (~ 2) is the g-factor, μ_B_ is the Bohr magneton, *J* (= 1/2 and 3/2) is the total angular momentum. For the second term, χ_vv_ indicates the van Vleck term. The values of *N* and χ_vv_ are summarized in Table 3. Provided that *N*_1/2_ and *N*_3/2_ are fixed to equal considering the local charge disproportionation model, the *M* data up to 60 T fit the Eq. (4). The best fits are shown by the dashed lines in Fig. 2c, with the fitting parameters given in Table 3. Our analysis suggests that ~ 20% of free spins (*J* = 1/2 and 3/2) are present. The orphan spins possibly emerged by the valence being off from tetravalent, which is no evidence from the crystal structural analysis. Although we cannot rule out other origins, these facts support that the PCD model is a good solution. As in the Ir^5+^ system, the PCD-generated isolated spins may be directly detected by XMCD measurements: it is a further issue. In addition, the van Vleck term was found to be more significant for SrLaGaRuO_6_.

**Table 3:** Results of fits to the isothermal *M* using the model described in the text. The parameters *N*_1/2_ and *N*_3/2_ are fixed to equal.

	${N}_{1/2}$ (Ru^5+^)	${N}_{3/2}$ (Ru^3+^)	*χ*_vv_ (cm^3^/mol Oe)
SrLaInRuO_6_	0.1088 (3)	0.1088 (3)	0.00869 (2)
SrLaGaRuO_6_	0.0937 (21)	0.0937 (21)	0.01503 (2)

The estimated van Vleck term of SrLaGaRuO_6_ is larger than SrLaInRuO_6_. According to Boltzmann statistics, the van Vleck term is proportional to the concentration of *J*_eff_ = 1 exciton. Therefore, the difference in χ_vv_ between SrLaInRuO_6_ and SrLaGaRuO_6_ is due to the different *Δ*. In the theoretical prediction, a non-cubic crystal field, generated by a distortion of the RuO_6_ octahedra, effectively reduces *Δ*^33^. Here, we introduce the bond angle variance σ, as a scale parameter of the polyhedral distortion. The σ-value in the RuO_6_ octahedra can be parametrized by the following formula,

5 $$\sigma =\sqrt{{\sum }_{i=1}^{12}\frac{{\left({\varphi }_{i}-{\varphi }_{0}\right)}^{2}}{m-1}}$$

where *m* is the number of O–Ru–O angles, φ_*i*_ is the *i*th bond angle of the distorted coordination-polyhedra, and φ_0_ is the bond angle of the coordination polyhedral with *O*_h_ symmetry; φ_0_ equals 90° for octahedron. Calculations using the atomic position parameters listed in Table 1 yield the σ-values of 7.7976° and 10.2708° for SrLaInRuO_6_ and SrLaGaRuO_6_, respectively, indicating a strikingly larger non-cubic crystal field in SrLaGaRuO_6_ than SrLaInRuO_6_. Therefore, the concentration of *J*_eff_ = 1 exciton of SrLaGaRuO_6_ should be larger than SrLaInRuO_6_, consistent with the large-small relationship of χ_vv_.

Furthermore, it is theoretically predicted that the SE interaction between *J*_eff_ = 0 reduces *Δ*. In SrLaInRuO_6_ and SrLaGaRuO_6_, the *J*_eff_ = 0 pseudospins interact via the SE interaction through Ru^4+^–O^2–^*M*^3+^–O^2–^Ru^4+^ paths with *M* = In, Ga. Thus, it is considered that the difference in the SE interaction between these two systems arises from the filled outermost orbitals, which are 4*d* and 3*d* orbitals for SrLaInRuO_6_ and SrLaGaRuO_6_, respectively. Therefore, it is reasonable that the SE magnitude is different.

Based on the results so far, it is reasonable to consider that the spin-glass transition in SrLaGaRuO_6_ is due to randomly arranged isolated spins. Strangely enough, however, no spin-glass transition has been observed in SrLaInRuO_6_, where the isolated spin concentration is comparable. However, it is unlikely that all the 19% localized spins interact strongly in SrLaGaRuO_6_ where the Ru–Ru distance is far apart. This fact suggests a difference in the magnitude of the interaction between randomly arranged isolated spins.

**Figure 3:**
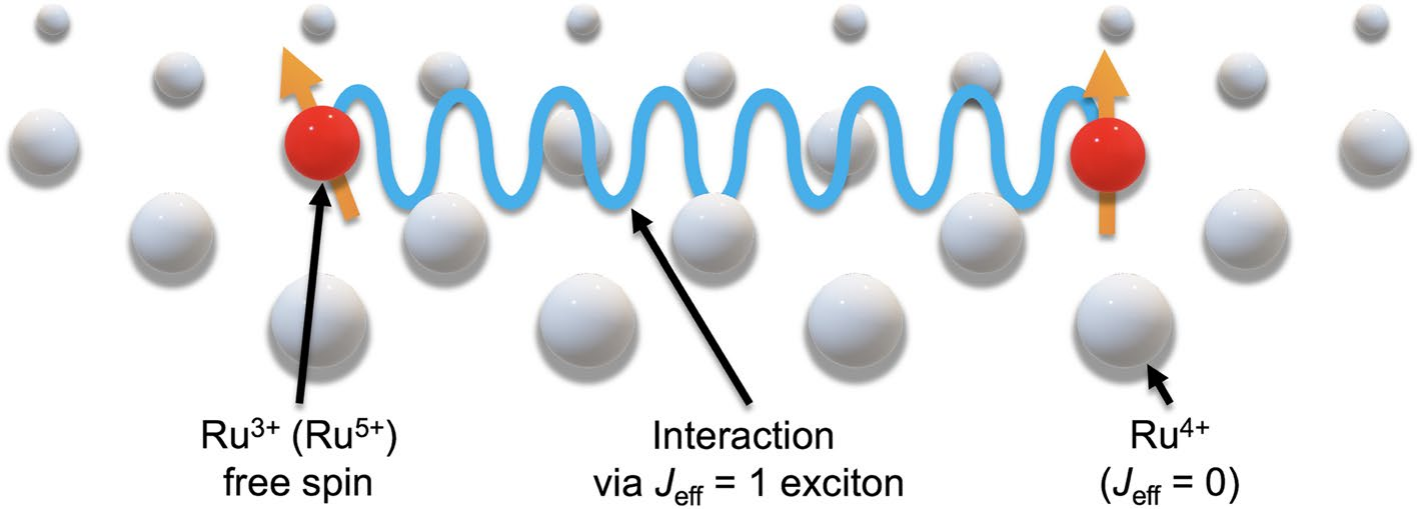
Schematic of the mechanism of spin-glass induced by isolated spin and *J*_eff_ = 1 excitons, as an analogy of a dilute magnetic alloy. The interaction between free spins mediated by mobile *J*_eff_ = 1 excitons corresponds to the RKKY interaction.

The origin of the spin-glass transition in SrLaGaRuO_6_ can be inferred by analogy with dilute magnetic alloys. In dilute magnetic alloys, partially arranged magnetic atoms interact with each other via RKKY interactions. As mentioned in the introduction, the *J*_eff_ = 1 excitons become a dispersive mode due to strong SE interactions^9^. In this situation, the mobile *J*_eff_ = 1 exciton may behave like a conduction electron. Therefore, the interaction via a mobile *J*_eff_ = 1 exciton between the free spins in a *J*_eff_ = 0 magnet can be regarded as an RKKY interaction. A schematic diagram of this mechanism is shown in Fig. 3. This interaction should be proportional to the concentration of *J*_eff_ = 1, which is consistent with the presence/absence of spin-glass transition. The feasibility of the spin-glass transition in the category of spin–orbit excitonic magnetism is very interesting and requires further theoretical studies. In the broad context, this finding also suggests that the several magnetic responses in *J*_eff_ = 0 magnets, which have been found so far, would be explained by the generated isolated spin model. Thus, we sincerely hope that it should be carefully re-examined.

## Summary

We have successfully synthesized new Ru^4+^ double perovskite oxides SrLaInRuO_6_ and SrLaGaRuO_6_. The temperature-dependent *M/H* and isothermal *M* data can be explained by the van Vleck magnetism of *J*_eff_ = 0 states with additional isolated spins possibly generated by the Ru^3+^/Ru^5+^ magnetic defects. While SrLaInRuO_6_ is paramagnetic down to 2 K, SrLaGaRuO_6_ shows spin-glass transition at *T*_f_ ~ 50 K. We propose that the origin of spin-glass is isolated spins couple via mobile *J*_eff_ = 1 excitons as an analogy of a dilute magnetic alloy. It is expected that the spin-glass transition due to the introduction of isolated spins demonstrates the existence of mobile *J*_eff_ = 1 excitons as dispersive modes as predicted in spin–orbit-entangled *d*^4^ ions.

## Data Availability

The datasets generated and analyzed during the current study are available from the corresponding author.
